# Stakeholders´ Perspectives on Riparian Zones Across Europe: Shared Views and Regional Contrasts

**DOI:** 10.1007/s00267-026-02542-w

**Published:** 2026-06-25

**Authors:** Giorgio Pace, Jose Barquin, Laura Concostrina-Zubiri, Luca Gallitelli, Maria Cristina Bruno, Monika Laux, Micael Jonsson, Massimiliano Scalici, Cláudia Pascoal, Ralf Schulz, Stefano Larsen

**Affiliations:** 1 Centre of Molecular and Environmental Biology (CBMA)/Aquatic Research Network (ARNET) Associate Laboratory, University of Minho, Braga, Portugal; 2 Institute of Science and Innovation for Bio-Sustainability (IB-S), University of Minho, Braga, Portugal; 3 IHCantabria, Instituto de Hidráulica Ambiental, Universidad de Cantabria, Santander, Spain; 4 Department of Sciences, University of Roma Tre, Rome, Italy; 5 Research and Innovation Centre, Fondazione Edmund Mach, Michele all’Adige, Italy; 6 National Biodiversity Future Center (NBFC), Università di Palermo, Palermo, Italy; 7 Institute for Environmental Sciences, RPTU – University of Kaiserslautern-Landau, Landau, Germany; 8 Department of Ecology and Environmental Science, Umeå University, Umeå, Sweden

**Keywords:** buffer zones, human-nature interactions, river management, biodiversity conservation, water quality

## Abstract

Riparian zones are transitional habitats linking terrestrial and aquatic ecosystems and connecting diverse socio-ecological landscapes within catchments. They naturally support high biodiversity and provide multiple ecosystem services, yet they have been extensively modified and degraded by human activities. Although Europe has established binding nature restoration targets, the absence of a standardised framework for riparian ecosystems across countries often leads to management inconsistencies, further complicated by differing stakeholder perceptions of threats and priorities. We surveyed stakeholders’ views on the ecological roles, threats, and management needs of riparian zones across five European countries (Sweden, Germany, Spain, Portugal and Italy). Responses from more than 500 participants show strong alignment between scientists and practitioners. Approximately half of all respondents considered their local river basins to be in low to moderate condition. Perceptions of key threats varied geographically: invasive species were viewed as a major concern in the Iberian Peninsula, whereas habitat modification was broadly recognized as a critical issue. Conversely, Swedish respondents viewed water-quality degradation as a minor threat. Management priorities also differed regionally, with German respondents frequently emphasising restoration, while Portuguese prioritised environmental education. Awareness of European eco-schemes supporting riparian restoration was generally low, particularly in Sweden. Ultimately, our large-scale survey reveals both shared and divergent stakeholder perspectives that mirror the environmental and ecological characteristics of riparian zones across Europe’s boreal, continental, Atlantic and Mediterranean ecoregions. These findings underscore the need to improve awareness of financial incentives and to strengthen support for riparian conservation and restoration across the EU.

## Introduction

Riparian zones represent the interface between terrestrial and aquatic ecosystems. They include the stream channel between low and high water level and the area of terrestrial landscape influenced by flooding and groundwater level fluctuations (Naiman and Décamps 1997). They are ecotones where biophysical conditions change rapidly over a small spatial extent, often supporting highly specialised species and significantly contributing to regional biodiversity (Sabo et al. 2005). These areas provide essential socio-economic benefits and ecosystem services (González et al. 2017), such as microclimate regulation (Bowler et al. 2012), filtering and trapping of pollutants, sediments and excess nutrients from catchment runoff (Dosskey et al. 2010), and flood protection by buffering, and slowing down floodwaters (Riis et al. 2020). Moreover, riparian habitats also offer recreational opportunities such as fishing, hiking, and birdwatching, contributing to human well-being and the aesthetic value of landscapes. Indeed, many riparian areas hold cultural, historical, and spiritual significance for local communities providing a unique sense of place and identity (Riis et al. 2020).

At the catchment scale, natural riparian zones form complex branching networks reflecting the geometry of streams and rivers (Benda et al. 2004). As such, they constitute ‘blue-green infrastructure’ that provide physical and functional connectivity among diverse habitats in the basin. However, riparian zones and floodplains have historically been areas of intense human activity, contributing to hydro-morphological and ecological disconnection between aquatic and terrestrial habitats (Singh et al. 2021; Urbanič et al. 2022). As a result, floodplains are among the most threatened and degraded ecosystems globally, especially in Europe, where less than 10% remain in relatively natural conditions (Janssen et al. 2021; Tockner et al. 2008).

Due to their high ecological connectivity, widespread distribution across the landscape and relatively small surface area, protecting and restoring riparian zones provide significant environmental benefits with minimal socio-economic drawbacks associated with land reclamation (González et al. 2017; Urbanič et al. 2022). Given the ambitious goals of the EU Biodiversity Strategy 2030 and the EU Nature Restoration Law (Hering et al. 2023), prioritizing the conservation and restoration of riparian areas presents a valuable opportunity to enhance biodiversity while simultaneously addressing socio-economic needs (Urbanič et al. 2022). However, assessment and management of riparian zones and floodplains still lack standardised and coordinated approaches across jurisdictions and countries. Also, statutory monitoring often focuses solely on aquatic biodiversity and water quality, overlooking the needs of non-aquatic organisms as quality elements (González del Tánago et al. 2021; González et al. 2017). This fragmented approach can lead to conflicts among stakeholders and contradictory policies. For example, efforts to improve flood protection may involve removing poplar and willow trees, which are valued for maintaining healthy alluvial forests in other contexts.

While the need for an integrated basin-scale management approach for conserving riparian zones is widely recognised, its practical implementation encounters many technical, ecological, socio-economic, and legal complexities (González et al. 2017; Hoppenreijs et al. 2024). For example, river basins often span multiple administrative boundaries and involve diverse stakeholders, including farmers, industries, and local communities, who may have competing interests. Achieving a balance between societal demands for resources and the critical ecological functions of riparian areas requires collaborative dialogue among all parties (Saklaurs et al. 2022; Gumiero et al. 2013). A crucial step in this direction is assessing the perspectives of diverse stakeholders on the use/engagement, conservation status, perceived functions, and identified threats to riparian ecosystems in their local areas. Because conflicting interests and a lack of agreement among stakeholders still represent barriers to successful ecological restoration (Cortina-Segarra et al. 2021), there is a clear need to better identify the unique and shared perspectives of major stakeholder groups across European countries. In this study, we gathered and analysed the views on local riparian zones among multiple stakeholders across five EU countries (Sweden, Germany, Spain, Portugal and Italy), reflecting a wide bio-climatic gradient that includes the Boreal, Alpine, continental, Atlantic and Mediterranean ecoregions. Specifically, we aimed at assessing similarities and differences in perspectives regarding the conservation status of riparian zones, their main ecological functions and threats, as well as the priority management actions and associated responsibilities. By tracing stakeholder views across these distinct dimensions, we seek to mirror the logical workflow of environmental managers, who first identify ecosystem functions, evaluate the threats endangering those functions, and ultimately priorities management actions based on these assessments.

In addition, we sought to appraise and compare specific stakeholders’ knowledge about riparian management and biodiversity, as ecological knowledge and policy awareness influence stakeholders’ willingness to support and adopt conservation measures. To this end, we examined their awareness of EU direct payment eco-schemes, a component of the Common Agricultural Policy (CAP), because these eco-schemes incentivise farmers and landowners to establish riparian buffers, a critical tool for improving stream health. Finally, we asked respondents to name any plant or animal species that they considered emblematic for their local riparian zones. By analysing these patterns, we aimed to evaluate stakeholders’ ecological familiarity with these ecosystems and determine whether their perception is linked to specialised riparian organisms or may reflect highly visible, generalist species. This link between policy awareness, biodiversity knowledge, and local perception can help reveal stakeholder expectations for conservation.

Participants represented around one hundred river basins and included representatives from academia, government agencies and nature conservation organizations, water and forestry managers, tourism entrepreneurs, as well as local citizens without professional involvement in river or riparian zone management. This also allowed us to examine if overall perceptions differed between scientists and practitioners.

## Methods

### Data Collection and Stakeholders Selection

**Fig. 1:**
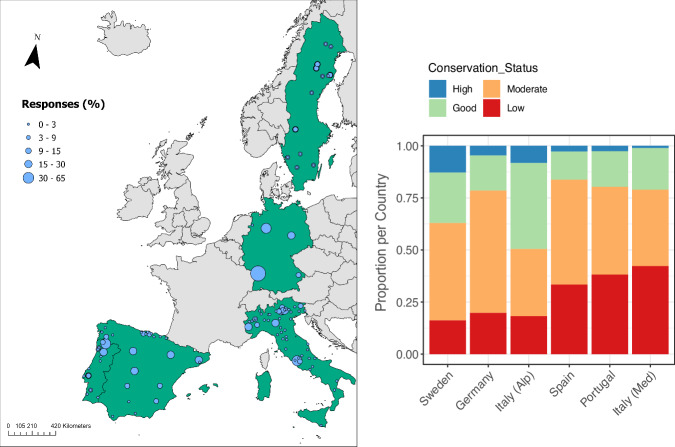
Map showing the distribution of respondents per Country. Right: perceived conservation status of the basins (Questions in Supplementary Materials: Section 3, Q7)

An online questionnaire was carried out in five European countries (Sweden, Germany, Spain, Portugal, and Italy; Fig. 1), using Google Forms (docs.google.com/forms). These countries were selected as key study areas for the Biodiversa+ project RIPARIANET (https://riparianet.eu/; Larsen et al. 2023), incorporating five European biogeographical regions: boreal, continental, Alpine, Mediterranean and Atlantic. Responses from Italy were categorised as either Mediterranean (from central and southern basins) or Alpine (northern mountain basins). We systematically identified the relevant stakeholder sectors, associations and target individuals based on existing literature and authors’ knowledge of the key social actors in each area. We sought to include multiple stakeholder groups within each country, by specifically reaching out for respondents within the following sectors: academics/research, agriculture, environmental education, landowners, nature conservation, fishing, forestry, government, environmental consulting, tourism and water management (Supplementary Fig. S1). Citizens with no professional involvement with riparian zones were also included. While every sector was successfully represented across all five countries, their relative proportions varied by region.

We shared the questionnaire by sending a personal email to each stakeholder contact using the native language of each country (i.e., German, Italian, Swedish, Portuguese, and Spanish).

### **Questionnaire Design**

This study employed a mixed-methods survey approach, incorporating both closed-ended and open-ended questions (Adams, 2015). Stakeholders were queried about key management and conservation challenges facing riparian zones within their respective countries. All survey procedures adhered to ethical guidelines, ensuring participant anonymity, informed consent, and the right to withdraw from the study without consequence (see Ethics statement). The questionnaire was structured into four sections (Supplementary Table S1). Section 1 (Q1–Q3) collected basic participant demographics, including age, gender, and relevant professional or personal experiences in relation to streams and riparian zones. Section 2 (Q4–Q5) assessed stakeholder perceptions regarding the primary environmental functions and threats associated with riparian zones. Respondents were asked to rank the perceived value of key ecological functions provided by riparian zones, including temperature regulation, nutrient buffering, habitat provision and flood protection. Similarly, they were asked to rank key threats to riparian zones in their area, including pollution, land use, invasive species, habitat modification and extreme climatic events. Section 3 (Q6–Q10) explored stakeholder perceptions of the overall conservation status of streams and riparian zones within their local basins/areas, qualitatively rated as poor, moderate, good and high. In addition, respondents were asked to name any plant or animal species they considered emblematic of local riparian zones. Section 4 (Q11–Q12) focused on identifying necessary management actions to enhance ecological integrity of riparian zones and the key stakeholders responsible for these actions.

The question about management priorities was open-ended, allowing respondents to describe the actions they considered vital to riparian conservation. To facilitate analysis, these qualitative responses were synthesized into a distinct set of key management actions, which were inductively developed from the participants’ suggestions (Table 1).

**Table 1:** Key terms synthesising the open responses about riparian management action priorities

Keyword	Definition of the mentioned management actions
Buffer	Increasing or maintaining distance of anthropogenic activities from the stream, and/or increasing the width of riparian vegetation.
Vegetation	Management of riparian vegetation, including planting native species, protection of riparian forests (e.g. avoid excessive clearing).
Banks	Actions to maintain or restore natural stream banks, also allowing overflow.
Flow and Barriers	Renovation or removal of barriers (e.g. dams, weirs), setting and restoring natural flow regimes.
Floodplains and Meandering	Increasing lateral connectivity between aquatic and terrestrial ecosystems, provide room for natural meandering and inundation dynamics.
Let it run wild	Limit direct interventions and allow rivers to re-establish natural dynamics.
Protection	Better and more effective conservation measures, including better and clearer environmental legislations.
Restoration	Specific and non-specific actions towards re-naturalisation of riverine habitats.
Invasives	Removing and limiting invasive species.
Pollution	Controlling and minimising inputs of pollutants, including WWTP and diffuse pollution from agriculture.
Sustainable use and Hydropower	Better implement the sustainable use of water resources and hydropower production.
Monitoring and Surveillance	Focus on increasing the environmental monitoring and control (including regulations and fines).
Awareness and Education	Increase sharing of information regarding riparian zones, its biodiversity and transfer of knowledge to land owners and the wider public.
I don’t know	Empty response, or direct statements of not knowing.

Finally, Q13 appraised whether respondents were aware of the EU incentives (eco-schemes) to establish riparian buffers along watercourses.

All responses were anonymised, and assigned a country-specific code (e.g., P = Portugal, Sp = Spain, Sw = Sweden, G = Germany, I = Italy). Representative quotes from participants are included in Supplementary Table S2.

### Ethics Statement

No institutional ethical clearance was required. However, the survey was implemented in accordance with the European Union´s General Data Protection Regulation (GDPR; https://gdpr-info.eu/). Specifically, three ethical principles were followed: (i) all respondents were fully informed about the scope, main goal of the research, and potential use of the data, as well as the dissemination of results; (ii) informed voluntary consent was obtained before participation, and (iii) anonymity and privacy were ensured for all interviewees. Participants had to agree to this consent form before they could fill in the questionnaire.

### Data Analysis

The results were described and analysed in terms of frequencies of responses for each country and overall. In order to quantify differences among countries and stakeholder types (e.g. academics vs practitioners) in terms of frequencies of responses, we used Chi-square tests on contingency tables, which are adequate for analysing survey data with different sample sizes among groups (Agresti 2002). All respondents with professional ties to riparian zones, but not identified as academics/researchers were considered practitioners. To help visualise patterns of significant differences, Pearson residuals were plotted showing the deviation of each specific answer from the overall expectations.

To examine differences among countries regarding the emblematic species mentioned by respondents, and to assess the emergence of underlying geographical patterns, we carried out a Principal Coordinate Analysis (PCoA) based on Bray-Curtis distance. All analyses were carried out in the statistical software R (R Core Team 2024).

## Results

### Respondents Characteristics Across Countries

We obtained 545 responses from multiple stakeholder types in each country (Supplementary Fig. S2). Across all countries, scientists and academics constituted ~30% of the participants, while practitioners working for government agencies and in nature conservation accounted for over 40%. Roughly 10% of respondents lacked direct professional ties to rivers or riparian ecosystems. The remaining participants included water and forestry managers, landowners, farmers, and people from the tourism sector, collectively representing a broad spectrum of interests within the “riparian community”. Most respondents were male (68%) in their forties and fifties. Each country and hence bio-geographic zone was well represented, largely reflecting differences in population size among countries: Sweden (11% of respondents), Germany (26%), Alpine Italy (17%), Mediterranean Italy (14%), Spain (20%) and Portugal (11%).

### General Perceptions of Riparian Zones’ Conservation Status, Functions and Stressors

Overall, respondents perceived local riparian zones as mostly in moderate (46%) and low (27%) conservation status (Fig. 1). Alpine Italy and Sweden were perceived as having relatively higher conservation status, while those in southern Europe (Portugal, Spain and Mediterranean Italy) the lowest scores. The perception of riparian conservation status was similar between practitioners and academics/scientists (Supplementary Fig. S2; Chi-square *p* = 0.13).

**Fig. 2:**
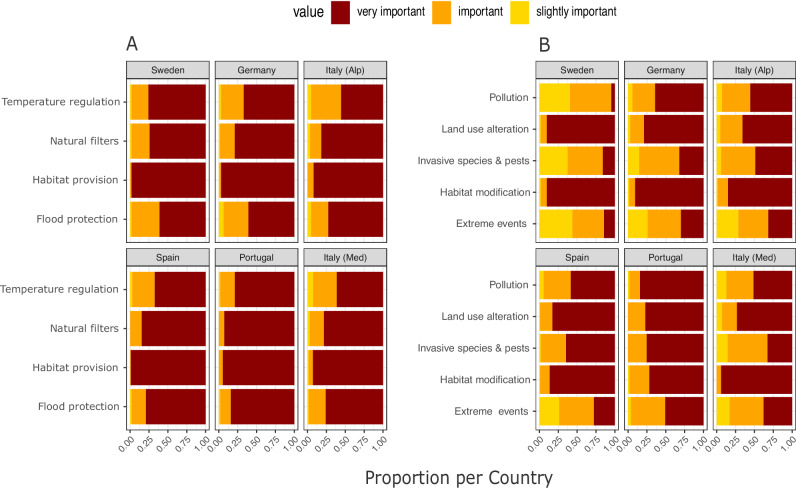
**A** Valued importance of key ecological functions of riparian zones (Questions in Supplementary Materials: Section 2, Q4). **B** Perceived importance of the main threats affecting riparian zones in each country (Questions in Supplementary Materials: Section 2, Q5)

Across countries, there was a broad agreement on the key ecological functions and services provided by riparian zones (Fig. 2A). Habitat provision for biodiversity was consistently indicated as the most important service, followed by the filtering function for pollutants and sediments from surface runoff. The perceived role of riparian areas for flood protection differed across countries (Chi-square = 24.4; df = 10; *p* < 0.01). Flood protection was considered very important by respondents in Spain, Portugal and Mediterranean Italy, but of lesser importance in Germany and Sweden.

In terms of perceived stressors to riparian zones, habitat alteration and land use were consistently seen as major concerns across countries. However, notable differences were also observed among countries (Fig. 2B). For example, degradation of water quality (‘Pollution’) was considered a minor concern in Sweden, whereas Portuguese respondents mentioned it as a key issue (Chi-square = 121; df = 10; *p* < 0.001). Similarly, invasive species were seen as a significantly greater threat to riparian zones in Portugal and Spain compared to Sweden and Germany (Chi-square = 108; df = 10; *p* < 0.001). On the same theme, the threat of extreme hydro-climatic events, such as floods and droughts was perceived as considerably higher in Portugal and Mediterranean Italy relative to other regions (Chi-square = 36.2; df = 10; *p* < 0.001).

### Perception of Emblematic Species

**Fig. 3:**
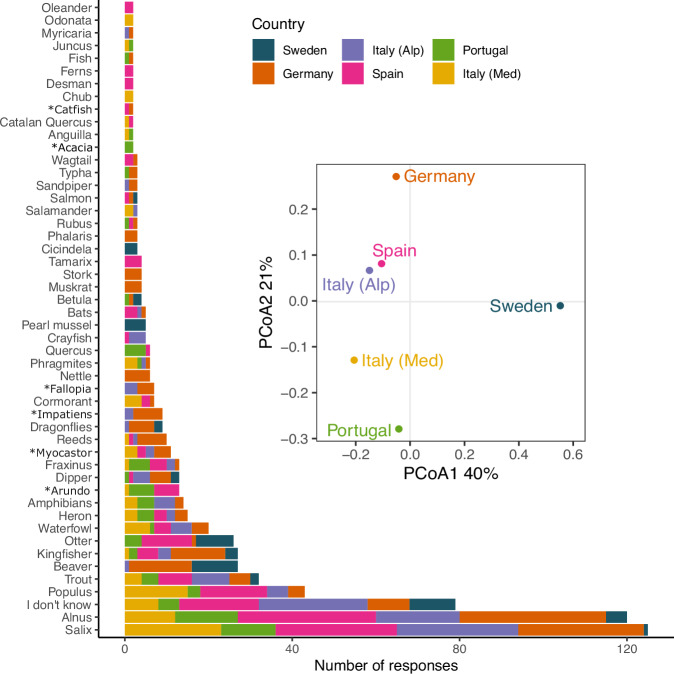
Histogram of emblematic species as mentioned by respondents. Insert shows the first plane of a PCoA based on species counts. Only species mentioned by at least two respondents were included (Questions in Supplementary Materials: Section 3, Q10). Note that the histogram reflects the raw total number of responses, which differs across countries. The taxon names are as reported by the respondents. *Non-native species (as far as could be determined from the taxon name provided by the respondent)

When asked to name emblematic species associated with local riparian zones, respondents mentioned a total of 124 species. Typical riparian trees, such as willows (*Salix* spp.), alders (*Alnus* spp.) and poplars (*Populus* spp.) were the most commonly mentioned. However, these trees were scarcely mentioned in Sweden, where animal species such as otters and beavers (the latter was also mentioned frequently in Germany) were more commonly cited. Several non-native species were also mentioned, including *Fallopia* sp., *Myocastor coypu* and *Arundo* sp. Surprisingly, a large proportion of respondents (mean: 40%, range: 15–80%) did not identify any species as emblematic of riparian zones in their respective areas. The distribution of those species mentioned by at least two respondents is shown in Fig. 3. A Principal Coordinate Analysis based on species counts formed a clear latitudinal gradient over the first two axes (explaining 40% and 21% of variation, respectively), with Germany and Sweden appearing relatively separated from the other countries (Fig. 3 insert). This is due to the relatively higher frequency with which German and Swedish respondents mentioned species such as beaver, otter and pearl mussel.

### Management Needs and Key Responsibilities

**Fig. 4:**
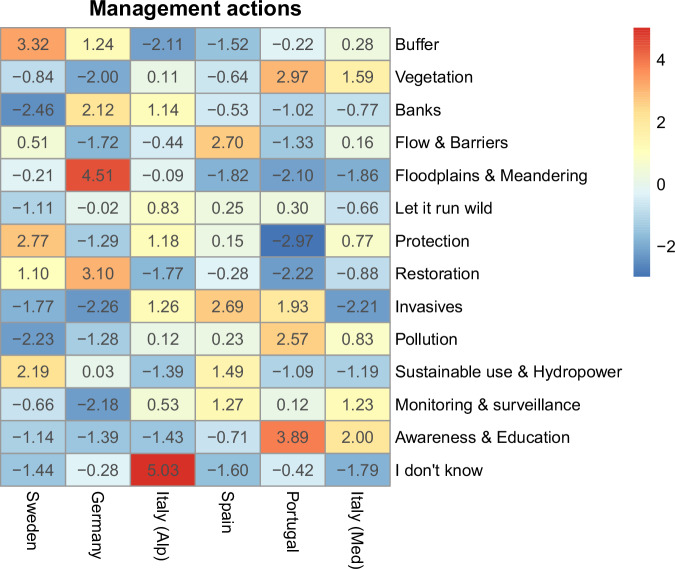
Chi-square Pearson residuals indicating deviation from expected frequency for each entry regarding management actions. Larger values indicate responses that were more frequent (red), or less frequent (blue) than expected (Questions in Supplementary Materials: Section 4, Q11)

Several management priorities were put forward by stakeholders to enhance the ecological conditions of riparian areas (Supplementary Fig. S3). Overall, restoration of habitats towards a more natural state (17%) was the most frequently cited strategy. Managing riparian vegetation and establishing buffer strips were also viewed as crucial by most respondents. Significant variations in perceived management priorities emerged across countries, as highlighted by the Pearson residuals of a Chi-square test (Chi-square = 254.5; df = 65; *p* < 0.0001; Fig. 4). In Sweden, the stakeholders emphasised the need to implement sustainable hydropower practices and the creation of riparian buffers. In Germany, restoration of river-floodplains connectivity and re-establishing the natural meandering course of rivers were viewed as a top priority. Respondents from the Iberian Peninsula emphasised the need to manage invasive species, reduce water pollution and restore longitudinal connectivity by removing barriers, such as hydropower dams and weirs. In Portugal and in Mediterranean Italy, respondents highlighted the importance of raising awareness and expanding opportunities for environmental education. In contrast, a relatively large proportion of respondents from Alpine Italy did not mention any specific management action (i.e., “I don’t know’).

Across countries, over 50% of respondents were reportedly unaware of the financial incentives (eco-schemes) offered by the EU Common Agricultural Policy (CAP) to support landowners in establishing vegetated riparian buffers (Supplementary Fig. S3). This lack of awareness was the most pronounced in Sweden.

**Fig. 5:**
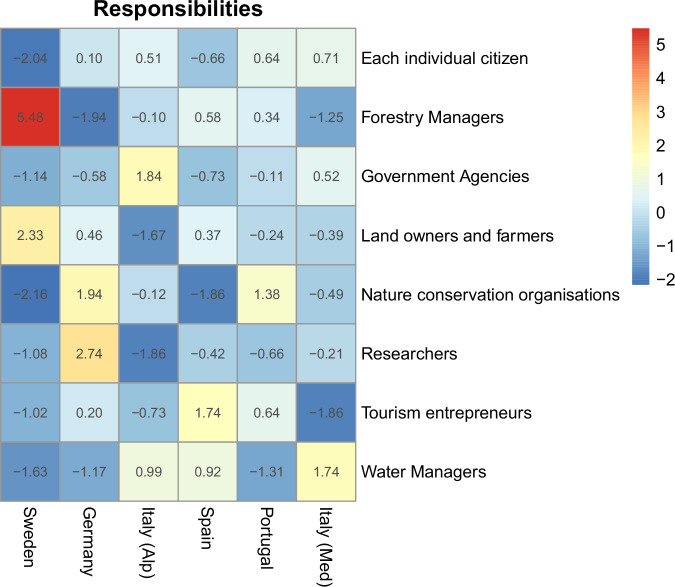
Chi-square Pearson residuals indicating deviation from expected frequency for each entry regarding management responsibilities. Larger values indicate responses that were more frequent (red) or less frequent (blue) than expected(Questions in Supplementary Materials: Section 4, Q12)

Overall, water managers, landowners, and government agencies were identified as having the greatest responsibilities for managing riparian zones (Supplementary Fig. S4). This perspective was shared by both scientists and practitioners (Supplementary Fig. S5). However, notable country-specific differences were observed (Chi-square = 102.2; df = 35; *p* < 0.001; Fig. 5). In Sweden, for instance, the key role of landowners in forestry and agriculture was emphasised, while in Germany scientists and academics were seen as strategic actors.

## Discussion

This study offers valuable insights into the perceptions of multiple stakeholder groups regarding the socio-ecological roles, threats, and management priorities of riparian zones across five European countries. We gathered responses from academics and practitioners working with or in riparian zones, as well as citizens without professional ties to rivers or riparian zones, resulting in a comprehensive assessment. Our findings reveal both commonalities and significant differences in stakeholder perspectives, reflecting the diverse bio-climatic contexts, socio-economic conditions, and cultural values across the studied regions.

### Conservation Status, Functions and Stressors

Overall, most respondents rated the conservation status of riparian zones in their areas as moderate to low - a view generally shared by both academics and practitioners. While this reflects the generally poor ecological conditions of riparian zones and floodplains in Europe (Grizzetti et al. 2017), notable differences across countries emerged. In particular, riparian zones in Sweden and Alpine Italy were perceived to be in relatively better condition than those in other countries. In Sweden, this perception is likely related to the extensive forest cover and limited agricultural activity throughout the region. This perception also aligns with the lower level of concern that Swedish respondents expressed regarding water pollution and invasive species. Similarly, in the Alpine region, streams and rivers at higher elevations drain less modified catchment areas and are generally perceived as more natural than downstream sections. However, it is worth mentioning that Alpine and peri-Alpine streams are often affected by alterations in the natural flow regime (e.g., hydropower schemes; Zolezzi et al. 2009), which may be less noticeable than changes in water quality or land use (Larsen et al. 2019).

Despite the poor ecological conditions reported across all countries, the majority of respondents acknowledged the fundamental importance of natural riparian zones for biodiversity conservation, particularly for providing habitat for a wide range of species. Similarly, the key role of riparian buffers in filtering sediment and nutrients from surface runoff was highly valued across countries. This shared view underscores the need for a strong focus on maintaining and restoring riparian habitats as a cornerstone of biodiversity conservation strategies across Europe (EEA 2019). Notably, the contribution of riparian areas to flood protection was highly valued by stakeholders from southern Europe (Portugal, Spain and Mediterranean Italy). This likely reflects the heightened sensitivity and exposure of these countries to climate extremes, such as severe droughts and floods (King et al. 2019), a view underscored by the high concern of hydro-climatic events raised by these southern regions. On this theme, our study also revealed significant variations across countries in the perceived threats to riparian ecosystems. While habitat modification due to human activities was consistently identified as a major concern across all countries, the emphasis posed on specific threats varied. Invasive species were perceived as a particularly pressing issue in the Iberian Peninsula, likely reflecting the prevalence of allochthonous plant species along riparian areas, such as *Acacia* spp., *Arundo donax*, *Phragmites australis*, *Fallopia japonica*, and *Eucalyptus* spp., which were introduced in Europe by the 19th century for various purposes and have since become widely spread (Jiménez-Ruiz et al. 2021; Nunes et al. 2019). For instance, the area dominated by *Acacia* species (e.g., *A. dealbata* and *A. longifolia*) increased by about 500 ha/year between 2005 and 2015 in Portugal (Ferreira et al. 2021). Conversely, water quality degradation was viewed as a less pressing concern in Sweden compared to other countries. This is likely because only about 7% of land surface is converted to agriculture in Sweden, with limited effects on overall water quality. Additionally, the population density is very low, especially in the northern half of the country, where many respondents were situated.

### Perception of Emblematic Species

Stakeholders and experts across countries mentioned a large number of plant and animal species that were associated with riparian zones, emphasizing stakeholders’ awareness of the biological diversity of these habitats (Bennett et al. 2014; Sabo et al. 2005). Remarkably, clear geographic patterns emerged from the ordination of these species counts on the first factorial plane. This suggests that local stakeholders’ informal perceptions of biodiversity reflects the actual distribution and abundance of species across European catchments.

Typical riparian trees were the most commonly named species (alders, poplars and willows), reflecting the recognised importance of native riparian forests as key structural elements of healthy riparian zones (Wohlgemuth et al. 2022; Dinca et al. 2025). Terrestrial native animals with tight connection to aquatic habitats were also frequently mentioned, including beavers, kingfishers and otters. However, several allochthonous species were also identified, such as *Fallopia japonica*, *Impatiens glandulifera* and the *Myocastor coypu*, which are widely distributed along streams and rivers across central Europe (Beerling et al. 1994; Vaissi and Rezaei 2023). The mention of these species as emblematic clearly highlights how common and widespread these have become along European waterways. From the survey, we are not able to assess if respondents actually perceived certain species as exotic or a threat. Notably, although German respondents mentioned *Fallopia* and *Myocastor*, they did not rank invasive species as particularly problematic in their area, as opposed to Spanish and Portuguese stakeholders.

It is interesting to note that many respondents did not mention any species at all. While this could simply reflect unwillingness to provide examples, it could also indicate a general lack of knowledge about riparian biodiversity, even among experts. This suggests that improving public knowledge of riparian biodiversity and fostering stakeholder engagement are key to promoting riparian conservation.

Overall, Sweden appeared to deviate the most from the other countries in terms of perceived emblematic species, likely due to a lack of invasive tree species in the riparian zone and a high abundance of alders and willows, making them less attractive as emblematic species (and hence scarcely mentioned by respondents). Conversely, the recent history of freshwater pearl mussels and beavers in Sweden influences the way they are perceived today. The freshwater pearl mussel has disappeared from 40% of its previously occupied localities in Sweden, in part because of changes in riparian and upland habitats (Degerman et al. 2013). This has led to continued population declines, although with considerable delay, due to the longevity of adult mussels. Hence, there are large efforts to protect and restore sites that still house the pearl mussel, likely resulting in this species being considered highly emblematic where present. The beaver was hunted to extinction in the late 1800s in Sweden, but after reintroduction in the 1920s, it has increased to its former range (Hartman 1995). Nevertheless, it is still considered relatively rare and emblematic in sites where it can be seen. It is noteworthy that beavers were also frequently mentioned in Germany, where their reintroduction in the last century has faced limited acceptance from the agricultural and forestry industries (Hohm et al. 2024).

### Management Needs and Key Responsibilities

Overall, despite country-specific variation, riparian restoration and protection were invoked as among the most important management priorities. This widespread agreement aligns with Europe’s growing commitment to ecosystem recovery, exemplified by the recently enacted Nature Restoration Law (European Commission 2022), which requires member states to develop restoration plans adapted to national context. In fact, our study also revealed notable differences in perceived management needs across countries, which in part reflect the specific values and threats associated with riparian zones by regions. For instance, tackling pollution, invasive species and managing riparian vegetation were seen as key priorities in the Iberian Peninsula, in line with the key perceived threats put forward by respondents in this region (Aguiar and Ferrera 2013; Muñoz-Mas et al. 2021).

In Sweden, tackling water pollution was not considered a key managing priority, in line with the limited concern regarding water quality issues demonstrated by respondents. Conversely, the maintenance of riparian buffers and protection of natural areas were regarded as top priorities. Sweden lacks dedicated legal protection for riparian forests, and a recent study revealed that forestry practices in the country often lead to fragmented and minimal buffer zones along waterways (Kuglerová et al. 2020). Also, hydropower is considered a main issue in Sweden, and not only because of its in-situ effects on aquatic habitats and organisms but also because of its impacts on riparian habitats. Because hydropower constructions have transformed most rivers and many larger streams in Sweden from free flowing to chains of run-of-river impoundments to meet energy demands, discharge does no longer follow seasonal patterns (Renöfält et al. 2010). As many riparian organisms depend on seasonal disturbance regimes for their persistence, especially in northern systems where seasonality is strong, hydropower threatens biodiversity in riparian habitats (Nilsson and Berggren 2000). Hence, focus has recently been on restoring the so-called environmental flows, mimicking natural discharge variation (e.g., Merritt et al. 2009; European Commission 2015). Similar restoration measures were also mentioned in Germany, where the focus was on floodplain restoration. This likely reflects the historical impact of river regulations and the increasingly acknowledged need for reconnecting rivers with their floodplains (Heyden and Natho 2022). Several recent restoration efforts in Germany provide exemplary success stories on this theme (Manfrin et al. 2020; Schulz-Zunkel et al. 2022).

Respondents from the Iberian peninsula, while rarely mentioning protection and restoration as management needs, clearly emphasised the importance of tackling invasive species and improving water quality, reflecting the concern about these threats raised by respondents in the region. Notably, stakeholders in Portugal emphasised the importance of rising awareness and environmental education, also among decision makers (da Silva 2017). Such emphasis was also put forward by stakeholders from Mediterranean Italy, possibly indicating an increasing pro-environmental standing in southern regions previously believed to be less sensitive to such issues (Gómez-Román et al. 2021). Additionally, this could reflect the increasing pro-environmental behaviour observed in aging populations (Wang et al. 2021).

Our survey also revealed that many stakeholders from Alpine Italy were unable (or unwilling) to provide management actions considered key for riparian conservation. This is surprising given the large proportion of respondents from the academic and nature conservation sectors. Considering that, overall, many respondents displayed limited knowledge about riparian biodiversity, these findings suggest the need for increasing environmental capacity building across different stakeholder sectors.

In the same vein, the apparent limited awareness of the EU eco-schemes designed to incentivise the creation and maintenance of riparian buffer zones among stakeholders across all countries is a concerning finding. This points to a significant gap in communication and knowledge dissemination regarding these important policies and likely reflects a limitation in how green incentives and policies are transmitted to local stakeholders across multiple administrative levels. Sweden, for example, exhibited a notably low level of awareness regarding EU eco-schemes, a surprising finding given that many respondents were employed by government agencies responsible for implementing such policies. This contrast is particularly striking as Sweden stood out among the surveyed countries for prioritising riparian buffer protection. Furthermore, Swedish respondents consistently assigned responsibility for riparian zone management to landowners in forestry and agriculture, the very sectors intended to benefit most from CAP incentives.

In terms of perceived responsibilities, landowners, water managers and government agencies were consistently identified as the key entities responsible for managing riparian zones; a view shared by practitioners and academics (Supplementary Fig. S4). However, differences across countries again reflect local needs and conditions. Swedish respondents emphasised the key role of forestry managers and land owners, a responsethat well aligns with the high priority given to the protection and establishment of buffer strips in the country. Conversely, stakeholders in Alpine Italy placed very limited management responsibility to local landowners, but rather towards water managers and government agencies. This may reflect the condition of many headwaters reaches in the Alps, where visible impacts are more often associated with concrete weirs, bank reinforcement and medium to large dams, rather than land-use modifications. Nonetheless, and in contrast to Sweden, issues related to hydropower production were not seen as a particularly important priority by stakeholders in Alpine Italy.

In contrast to other countries, German respondents stressed the key role of academics in the management of riparian zones. While it is unlikely for scientists to directly manage natural resources, these responses emphasise the perceived importance of generating scientific evidence and knowledge in guiding decision making in nature conservation (Rodríguez-González et al. 2022).

## Conclusions and Policy Recommendations

In this study, we provide a broad picture of the values, threats and management needs of riparian zones from the perspective of multiple stakeholders in Europe. We highlighted both commonalities and differences in stakeholder perceptions, which reflect the diverse ecological conditions of streams and riparian ecosystems included in the survey. A common view among the majority of respondents is that riparian zones are key for supporting regional biodiversity, yet they are perceived to be in relatively poor conservation condition. Country-specific differences can be associated with the large bio-climatic gradient and local-scale differences across our study catchments, reinforcing the importance of context-specific approaches to riparian ecosystem management. These insights have significant implications for the successful implementation of the European Nature Restoration Law, as well as the CAP in the coming years. Effective policy implementation will require comprehensive assessments of both ecological needs and culturally-driven preferences.

The unexpectedly low awareness of the CAP among stakeholders highlights critical gaps in one of the EU´s largest and most costly policies, accounting for about one third of the 2023-2027 budget. This lack of awareness could directly hinder the achievement of CAP objectives that rely on healthy riparian zones, such as biodiversity conservation and climate change mitigation. Taken together these findings could help bridge the gap between policy and practice. For instance, national and regional agricultural agencies should improve how environmental incentives are shared and communicated; the widespread lack of awareness surrounding CAP financial schemes demands direct outreach and practical workshops co-designed with local farming and forestry unions. Second, river basin authorities and regional environmental protection agencies could deliver guidelines for context-specific ‘Riparian Action Plans’. For instance, in the boreal region, such as Sweden, authorities should work directly with private landowners to enforce mandatory, unharvested forest buffers that protect streams from logging impacts. Conversely, in southern European catchments, managers and local conservation groups could stimulate community-led programs targeting invasive species eradication and water quality improvement (Cortés-Avizanda et al., 2022).

Considering the broad bio-climatic gradient covered by this survey, responses can be interpreted within a space-for-time approach in view of future climatic change. For instance, the issues and challenges highlighted by southern countries could offer insights on how perspectives might evolve at higher latitudes as temperatures rise or as invasive species migrate toward higher latitudes. Potentially, this might prepare practitioners and managers of riparian zones in northern regions for future impacts and thereby enable more effective management plans for riparian conservation in the future.

While the participation of stakeholders is crucial for fostering public support towards government decisions, conflicts among professionals may arise regarding specific management actions and objectives. Although our survey was not explicitly designed to evaluate such conflicts, it suggests that practitioners and academics largely agree on the main threats and responsibilities related to riparian ecosystem management. This is a promising finding, which could help facilitate dialogue among expert groups. However, further efforts are needed to involve policy decision-makers in similar large-scale surveys to capture a policy-oriented perspective alongside technical opinions.

## Supplementary information

Supplementary information

## Data Availability

The data are available upon request.
