# Supplementary material for: Stakeholders´ Perspectives on Riparian Zones Across Europe: Shared Views and Regional Contrasts
Source: Environ Manage. 2026 Jun 25;76(7):226. doi: 10.1007/s00267-026-02542-w (PMC13303545; doi:10.1007/s00267-026-02542-w)
Supplement: Supplementary file 1 — Supplementary information [file 267_2026_2542_MOESM1_ESM.docx]

**Supplementary Information**

**Stakeholders perspectives on riparian zones across Europe: shared views and regional contrasts**

Giorgio Pace * (1,2), Jose Barquin (3), Laura Concostrina-Zubiri (3), Luca Gallitelli (4), Maria Cristina Bruno (5,8), Monika Laux (6), Micael Jonsson (7), Massimiliano Scalici (4,8), Cláudia Pascoal (1,2), Ralf Schulz (6), Stefano Larsen (5,8)

(1) Centre of Molecular and Environmental Biology (CBMA) / Aquatic Research Network (ARNET) Associate Laboratory, University of Minho, Braga, Portugal.

(2) Institute of Science and Innovation for Bio-Sustainability (IB-S), University of Minho, Braga, Portugal.

(3) IHCantabria, Instituto de Hidráulica Ambiental, Universidad de Cantabria, Santander, Spain.

(4) University of Roma Tre, Department of Sciences, Viale Guglielmo Marconi, 446 00146 Rome, Italy.

(5) Research and Innovation Centre, Fondazione Edmund Mach, Via Mach 1, 38098 S. Michele all’Adige, Italy.

(6) Institute for Environmental Sciences, RPTU – University of Kaiserslautern-Landau, Landau, Germany.

(7) Department of Ecology and Environmental Science, Umeå University, Umeå, Sweden.

(8) National Biodiversity Future Center (NBFC), Università di Palermo, Piazza Marina 61, 90133 Palermo, Italy.

**Keywords:** stakeholders survey, human-nature interactions, river management, riparian forests, water quality, environmental policy?

*Corresponding author contact: giorgio.pace@bio.uminho.pt

**Table S1. Sections and questions of the online questionnaire.**

| **N°** | **Theme** | **Question** |
| --- | --- | --- |
| Q1 | Personal Information | What kind of activity brings you in contact with the river and its riparian zone? |
| Q2 |  | Which of the following categories best suits your professional activity in the river and riparian zone? |
| Q3 |  | Which of the following activity(ies) do you undertake on or along rivers? |
| Q4 | General Perception of Riparian Zone | Which, in your opinion, are the most important environmental benefits that riparian zones provide? |
| Q5 |  | What are, in your opinion, the most important threats for the riparian zones? |
| Q6 | River/Riparian Zone  Conservation Status | Please specify the river basin you are most familiar with |
| Q7 |  | What is, in your opinion, the overall conservation status of the riparian zones in your basin/area? |
| Q8 | Threats | Please indicate which location (river and closest landmark/village) where, in your opinion, riparian zones have the BEST conservation status in your river basin. |
| Q9 |  | Please indicate which location (river and closest landmark/village) where, in your opinion, riparian zones have the WORST conservation status in your river basin. |
| Q10 |  | What is, in your opinion, the most characteristic species (plant and / or animal) associated with riparian zones in your river basin? |
| Q11 | Management of Riparian Zone | In your opinion, what should/could be done to increase the ecological integrity/improve the condition of the riparian zone in your area? |
| Q12 |  | In your opinion, which of the following categories are most responsible and could do more to protect your riparian zones? |
| Q13 |  | Are you aware that under the European Common Agricultural Policy (CAP), farmers and landowners are entitled to incentives and economic benefits to establish vegetated riparian buffers along running water? |

**Table S2*.* Selected specific respondents for key topics.**

| **Theme** | **Selected respondents** |
| --- | --- |
| River/Riparian Zone  Conservation Status  &  Threats | P1: “In the Cávado river basin there are some places that due to industry end up suffering the consequences, pollution, sand drainage, improper cutting of riverine vegetation, intensive agriculture (Gilmonde/Mariz, Manhente/Barcelinhos, Ruães/ Padim da Graça)”  P2: “Mira canals, close to Casal de S. Tomé, Carro Meu and other small towns. The water lines are heavily modified to control flow, the banks are also heavily modified to facilitate access and there is a lot of agricultural activity near them.”  P3: Tejo (due to urbanization, absence of riparian corridors)”  G1: “I consider the fully channelized sections within Wissembourg and the stretch of the Lauter from Berg to Neuburg (the mouth of the Lauter) to be the least well-preserved sections due to the strong straightening and direct use of the riparian zone (urban areas and agriculture)”  G2: “Berg, Lauter is straightened and built-up, there are no riparian strips.”  G3: “For example, the Danube Canal. Morphologically speaking, this is the most heavily built-up tributary/branch of the Danube. Other streams that feed into the Danube (e.g., Alsbach) are completely underground. There are efforts to partially lead them above ground again, although it is questionable how much positive benefit this brings to the ecosystem as a whole if only slightly affected islands exist along slightly affected stretches”  I1: “From Tirano upwards: steep banks; shallow water and/or constantly fluctuating levels due to the dams; impossibility for fish to migrate; concrete everywhere”  I2: “Riparian habitats adjacent to private residences or used for unregulated sport fishing. Causes: removal of native riparian vegetation, planting of ornamental plants, littering-leading to habitat alteration and easier entry of invasive exotic species. These areas are difficult to access and therefore hard for authorities to monitor."  I3: “Po River, High section, municipality of Crissolo. Completely artificial riparian zone, straightened river, plus a series of weirs due to urbanization in the valley floor during the economic boom.”  Sw1: “Probably the final stretch of the Saja-Besaya, due to existing physical alterations, invasive species and plastic pollution”  Sw2: “The most upper parts, because of intense forestry and narrow buffer zones from clearcuts”  Sw3: ” at the hydropower dam in Stornorrfors”  Sp1: ”The surroundings of Torrelavega due to the channelling and discharge of industrial and urban wastewater”  Sp2: “Corneja Valley, Moros River and Becedas River (they are in areas with greater human pressure and lower level of protection)”  Sp3: “Rivers in the east of Alicante, because many are temporary, are not perceived as an ecosystem to be conserved. Example of the Vinalopó River.” |
| **Theme** | **Selected respondents** |
| Riparian Zone  Management | P1: “Raise awareness with riverside owners and the general public, increase monitoring and control”  P2: “Firstly, civil education is the factor that weighs the most… Therefore, in my opinion, it never hurts to carry out awareness-raising actions at the local level, to raise awareness of the problems that certain actions may cause.”  P2: “Educate public to not leave litter and carry out organized actions of river and forest cleaning”  G1: “Removal of weirs or conversion to permeable weirs without fish hazards. Creation of flood areas, dissolution of rigid structures”  G2: “Enlargement of the floodplain area, as well as enabling more natural floodplain dynamics”  G3: “Enlarge the edge strips to increase biodiversity and minimize the input of pesticides, as large parts of the Lauter are used for agriculture “  It1: “Restore lateral connectivity, reopen oxbow lakes and floodplains to the river, and allow them to flood even outside of hydrological emergencies. This would have a beneficial effect on many animal and plant species. The river cannot be seen merely as a channel for distributing water or extracting sand and gravel. It is much more than that, and riparian habitats are one of its crucial components. Hundreds of millions of euros have been allocated for the renaturation of the Po, yet the focus remains solely on sand extraction and emergency management. Nothing serious is being done for biodiversity!"  It2: “Increase the continuity of riparian vegetation, enhance the level of protection, and raise public awareness about the ecological functions of the riparian area.”  It3: “Give more managerial power to naturalistic research groups and environmental protection agencies."  Sw1: “ Scrutinize hydropower”  Sw 2: “ Change legislation connected to forestry and agriculture” |

**Supplementary Figures**

**
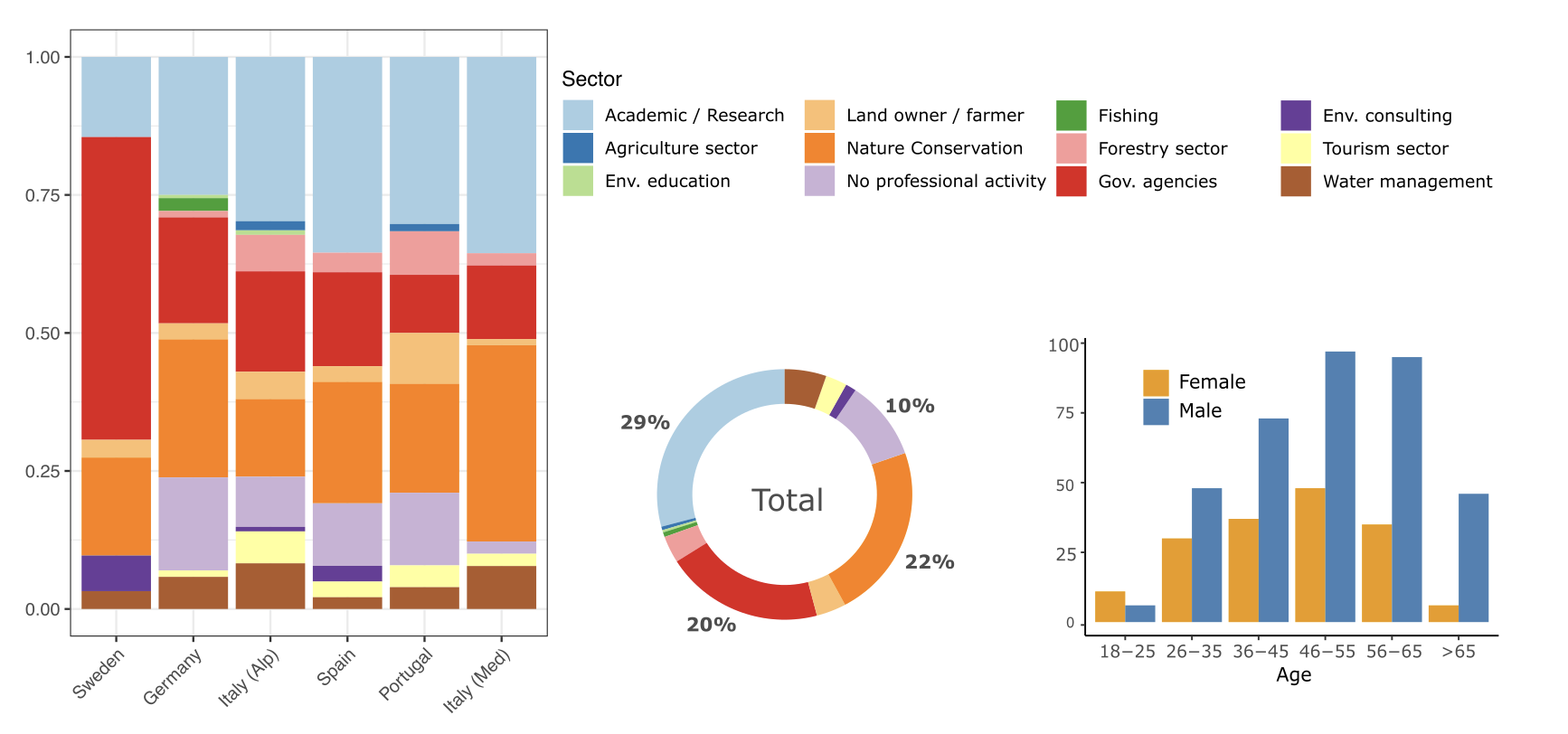
**

**FIGURE S1 - Main sector and age profile of the respondents. Four respondents did not declare gender identity (Questions in Section 1, Q1-Q3).**


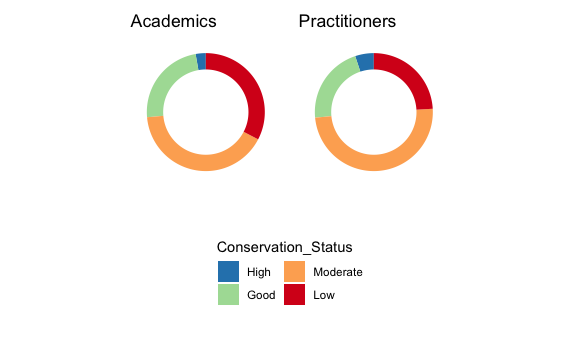


**FIGURE S2. Similarity in perception of ecological status between scientists and practitioners.**


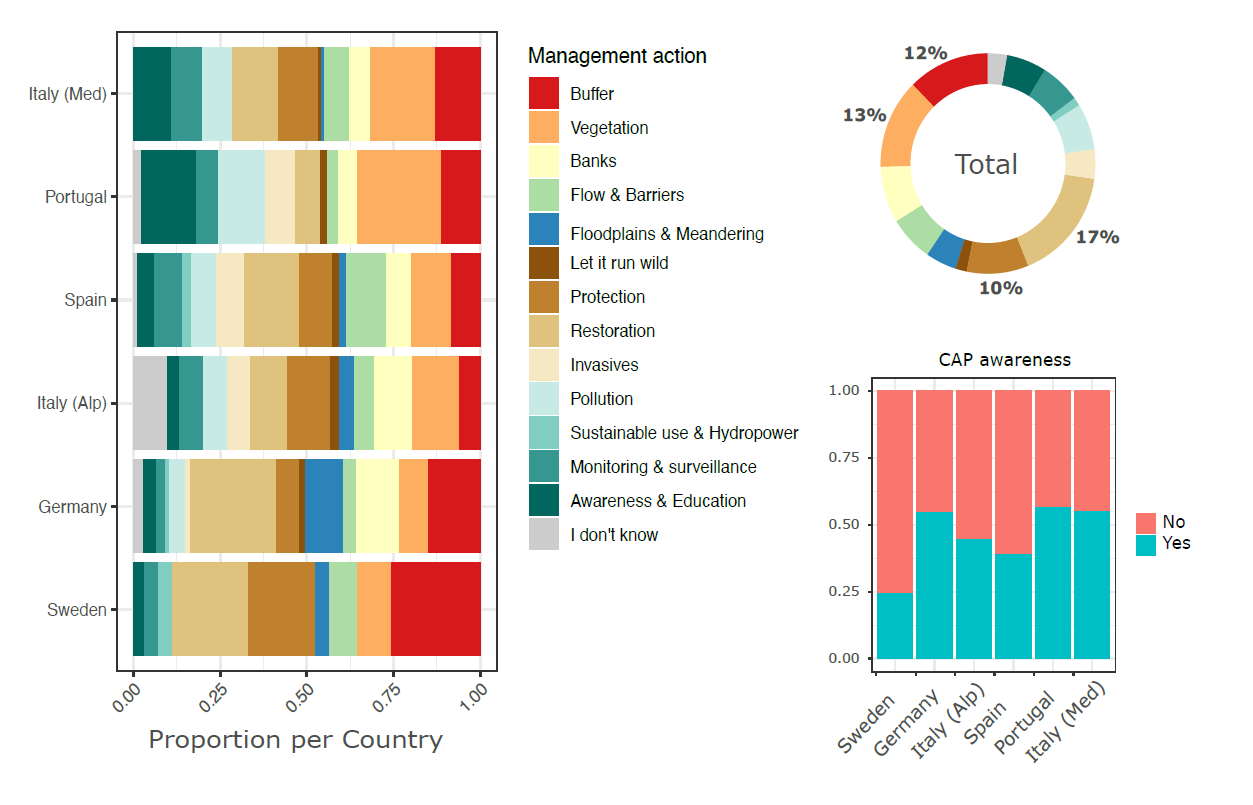


**FIGURE S3. Management action priorities mentioned by stakeholders, per country and in total,  and their awareness of the CAP financial incentives for buffer zones.**


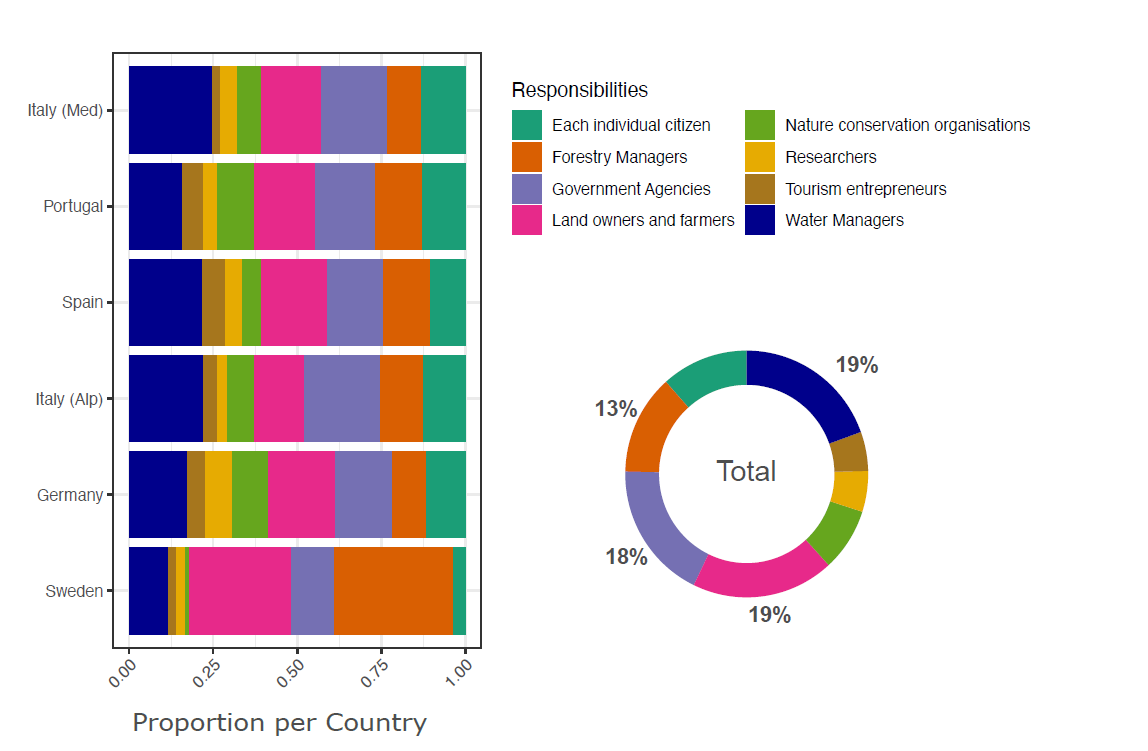


**FIGURE S4. Key responsibilities for the management of riparian zones, as mentioned by stakeholders, per country and in total.**


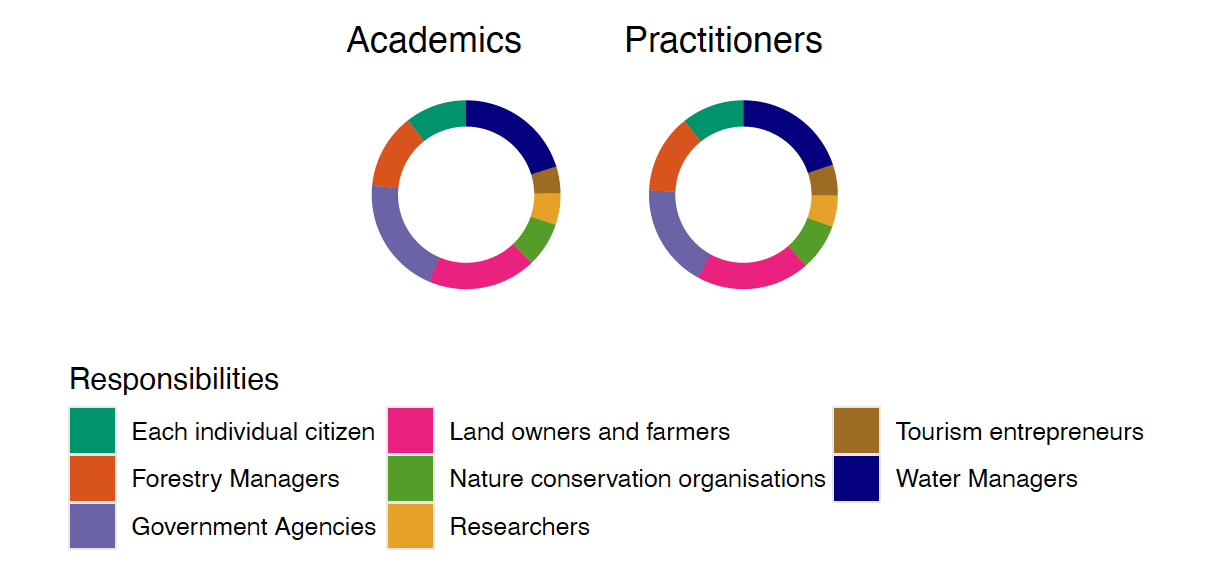


**FIGURE S5. Similarity in perception of key responsibilities between scientists and practitioners.**
